# Glucocorticoids in acute pancreatitis: a propensity score matching analysis

**DOI:** 10.1186/s12876-021-01907-1

**Published:** 2021-08-25

**Authors:** Meng Wang, Zongxing Jiang, Hongyin Liang

**Affiliations:** 1 Department of Traditional Chinese Medicine, General Hospital of Western Theater Command (Chengdu Military General Hospital), Chengdu, China; 2 Department of General Surgery, General Hospital of Western Theater Command (Chengdu Military General Hospital), Chengdu, 613000 China

**Keywords:** Glucocorticoids, Acute pancreatitis, Hypertriglyceridemic, Propensity score matching analysis

## Abstract

**Background:**

There are few reports about the effect of glucocorticoids in the treatment of acute pancreatitis in humans. This study aims to evaluate the effect of glucocorticoids in the treatment of acute pancreatitis by propensity score matching analysis.

**Results:**

Acute pancreatitis patients admitted between 2014 and 2019 were collected from the database and analyzed. Included patients were divided into the glucocorticoids-used group (GC group) and the non-glucocorticoids-used group (NGC group) according to whether glucocorticoids were used. A total of 818 eligible patients were included in the final analysis. Seventy-six patients were treated with glucocorticoids, and 742 patients were treated without glucocorticoids. Before propensity score matching, the triglyceride levels (38.2 ± 18.5 vs. 20.2 ± 16.8, *P* < 0.05) and Acute Physiology and Chronic Health Evaluation II (APACHE II) scores (7.1 ± 2.5 vs. 4.5 ± 2.1, *P* < 0.05) at admission were significantly higher in the GC group than in the NGC group. The incidence of multi-organ failure (33.3% vs. 11.9%, *P* < 0.05) was significantly higher in the GC group than in the NGC group. Patients in the GC group showed a positive balance of fluid intake and output over 72 h. After 1:1 propensity score matching, 59 patients from each group (GC and NGC) were included in the analysis. There were no significant differences in age, sex, body mass index, triglycerides, or APACHE II scores between the two groups (*P* > 0.05), and the patients’ clinical outcomes were reversed. The proportion of patients with organ failure (40.7% vs. 52.5%, *p* < 0.05) and multi-organ failure (35.0% vs. 67.7%, *P* < 0.05) was significantly lower in the GC group than in the NGC group. Furthermore, patients in the GC group had significantly shorter lengths of hospital stay (12.9 ± 5.5 vs. 16.3 ± 7.7, *P* < 0.05) and costs (25,348.4 ± 2512.6vs. 32,421.7 ± 2813.3, *P* < 0.05) than those in the NGC group.

**Conclusions:**

This study presents preliminary confirmation of the beneficial effect of glucocorticoids in the treatment of acute pancreatitis. More high-quality prospective studies are needed in the future.

## Introduction

Acute pancreatitis (AP) is a commonly occurring acute abdominal disease with multiple causes [1]. Severe acute pancreatitis (SAP) may be life-threatening and result in serious economic and health burdens [2]. During the early stage of AP, the systemic inflammatory response and subsequent multi-organ impairment are the most significant manifestations, resulting in the first peak of death in patients with AP [3, 4].

Glucocorticoids (GCs) are broad-spectrum inflammation-suppressing drugs that exert a broad range of anti-inflammatory effects [5]. GCs are versatile and may lead to some adverse effects, such as hyperglycaemia, secondary infections, osteoporosis, wound healing difficulty, and gastrointestinal bleeding [6]. At present, the effects of GCs in asthma, chronic obstructive pulmonary disease (COPD), and systemic vasculitides are well-acknowledged [7]. GCs are also considered to have beneficial, though debatable, effects in a series of other inflammatory diseases, including sepsis shock [8], community-acquired pneumonia [9], burns [10], and acute respiratory distress syndrome (ARDS) [11]. Recently, it has been reported that patients with COVID-19 may also benefit from the use of GCs to counter inflammatory storms [12, 13].

The role of GCs in the treatment of AP has been studied for a long time [14, 15]. In animal models of AP, the use of GCs has shown favorable results [16, 17]. However, there are few reports about the effect of GCs in the treatment of AP in humans. In this study, we used the propensity score matching (PSM) analysis to re-examine the contribution of GCs in the treatment of AP.

## Materials and methods

### Study design

This is a retrospective single-center cohort study. This study was performed following approval from the ethics committee of the General Hospital of Western Theater Command (GGW2019017) and conducted in accordance with the Declaration of Helsinki.

We utilized a database that recorded the information of AP patients admitted to our hospital since 2012 to conduct this retrospective research. All patients in this database between January 2014 and December 2019 were screened. Eligible patients were 18–70 years old, diagnosed with hypertriglyceridemic AP, and admitted within 48 h of onset. AP patients complicated with immune deficiency, pregnancy, or malignancy were excluded from this study. Patients included were divided into the glucocorticoids-used group (GC group) and the non- glucocorticoids-used group (NGC group) according to whether GCs were used.

The diagnosis and severity of AP were determined based on the 2012 Atlanta criteria [1]. The identification of hypertriglyceridemic AP was performed according to previous studies, and the serum triglyceride (TG) levels of patients with hypertriglyceridemic pancreatitis were usually above 11.3 mmol/L [18].

### Data collection

The data were collected from this database of AP patients. All data collectors were blinded to the study aims being investigated at the time of data abstraction. All medical records were reviewed by another independent physician. Inconsistent scores were recalculated until the same scores were achieved.

Demographic information, including age and sex, was collected. Some valuable evaluations were also performed. For example, Acute Physiology and Chronic Health Evaluation (APACHE II) scores [19] and Sequential Organ Failure Assessment (SOFA) scores [20] were collected to assess the severity of AP. The APACHE II and SOFA scores were calculated using the worst parameters during the initial 24 h after admission. Serum TGs at admission and body mass index (BMI) were also recorded. The collected therapeutic outcome indicator data included mortality, prevalence, duration of organ failure, the proportion of patients requiring further intervention, the incidence of peripancreatic necrosis infection and gastrointestinal bleeding, length of stay (LOS), and hospital costs. Several indicators were also evaluated to estimate the impact of GCs administration on fluid resuscitation. Inflammatory indicators, such as C-reactive protein (CRP) and tumor necrosis factor-α (TNF-α), at 1 week after hospitalization were also measured.

### Management of AP

Initially, all patients were treated conservatively as recommended in the guidelines [21, 22]. A revised step-up approach was used to manage AP-associated complications, including ascites, acute peripancreatic fluid collection, acute necrotic collection, and infected necrosis [23]. Prophylactic antibiotics are not routinely utilized. If the patient has symptoms of systemic infection, as well as elevated temperature, increased blood count, or positive blood cultures, antibiotics may be administered as appropriate.

Until now, there is no evidence to support the conventional utilization of GCs in AP. GCs are only used in some AP patients with severe inflammatory responses in the early stages of AP in our center. For example, the patients with hemodynamic instability (mean arterial pressure ≤ 65 mm Hg), severe acute respiratory distress (PaO2/FiO2 ≤ 100 mmHg), persistent organ failure (> 24 h), or multi-organ failure within the first 48 h of onset were considered for GCs treatment. The results of such a scheme included: (1) Patients treated with GCs tended to be more severely; (2) Not every patient with severe disease was treated with GCs. Therefore, in this retrospective study, we grouped based only on whether GCs were used. The PSM analysis was used to match the baseline between two groups, and thus further analysis was performed, which is not conducted in previous researches. The major types of GCs used were prednisone, dexamethasone, and hydrocortisone. The duration of GCs used was 1–6 days. The doses of prednisone or the equivalent doses of other glucocorticoids are usually less than 80 mg/d.

### Propensity score-matching analysis

PSM analysis was performed in this study with the matching package in R software (version 4.0.2 for Windows, Bell Laboratories) and conducted with the 1:1 nearest neighbor matching method. The covariates included sex, age, BMI, TGs, APACHE II scores, and SOFA scores.

### Statistical analysis

Categorical variables are described using frequencies and percentages. Continuous variables are summarized as medians (quartiles) or mean values (± SDs) when appropriate. Pearson χ2 test or Fisher’s exact test was used to determining the association between categorical variables. The student’s t-test or the Wilcoxon rank-sum test was used for continuous data as appropriate. Analysis of variance (ANOVA) or the Kruskal–Wallis test was used for comparisons between more than two groups, as appropriate. Statistical analysis was performed using SPSS, version 20.0 for Windows (SPSS Inc., Chicago, IL). *P* < 0.05 was considered statistically significant.

## Results

From January 2014 to December 2019, a total of 3478 patients were admitted to our hospital and documented in the database. Among these patients, 818 patients who met the inclusion and exclusion criteria were included in the final analysis. Seventy-six patients were treated with GCs, regardless of the type or amount of GCs used or the duration of GCs use.

### The baseline characteristics were matched via PSM analysis

The baseline characteristics of the patients are shown in Table 1. Before propensity score matching, the serum TGs (38.2 ± 18.5 vs. 20.2 ± 16.8, *P* = 0.005 < 0.05) and APACHE II scores (7.1 ± 2.5 vs. 4.5 ± 2.1, *P* = 0.034 < 0.05) at admission were significantly higher in the GC group than in the NGC group. The SOFA score (4.4 ± 1.5 vs. 2.1 ± 1.6, *P* = 0.063) was higher in the GC group than in the NGC group, although the difference was not statistically significant. These results suggest that patients in the GC group had a higher severity of disease at admission than those in the NGC group. After 1:1 propensity score matching, 59 patients from each group (GC and NGC) were enrolled in the analysis. There were no significant differences in age, sex, BMI, TGs, APACHE II scores, or SOFA scores between the two groups.

**Table 1:** Patient Characteristics at Baseline

Characteristic	Original cohort (n = 818)		*P*	Matched cohort (n = 118)		*P*
	GC group (n = 76)	NGC group (n = 742)		GC group (n = 59)	NGC group (n = 59)	
Age, year	45.1 ± 14.3	42.3 ± 16.6	0.429	44.9 ± 15.3	44.1 ± 16.1	0.894
Sex, Male, n (%)	44 (57.89%)	385 (51.89%)	0.132	33 (55.93%)	34 (57.62%)	0.813
BMI, kg/m^2^	28.1 ± 4.8	25.7 ± 3.9	0.107	27.2 ± 4.9	27.0 ± 4.1	0.927
TGs, mmol/L	38.2 ± 18.5	20.2 ± 16.8	0.005*	37.1 ± 17.9	37.51 ± 17.75	0.798
APACHE II score	7.1 ± 2.5	4.5 ± 2.1	0.034*	7.2 ± 2.4	7.1 ± 2.3	0.829
SOFA score	4.4 ± 1.5	2.1 ± 1.6	0.063	4.3 ± 1.5	4.3 ± 1.5	0.968

Abbreviation: BMI, Body mass index; TGs, Triglycerides; APACHE II, Acute Physiology and Chronic Health Evaluation II; SOFA, Sequential Organ Failure Assessment

*Significant difference

### The comparison of the clinical outcomes between two groups

The clinical outcomes of the two groups are shown in Table 2. Before propensity score matching, there was no difference in mortality between the two groups (3.9% vs. 2.4%, *P* = 0.676). In the GC group, 2 patients died of severe secondary infection of peripancreatic necrosis and 1 patient died of organ failure in the early stage of AP. In the NGC group, the major cause of death is also the secondary infection of peripancreatic necrosis (n = 11). The other cause of death included pulmonary Infection (n = 3), peripancreatic necrotic hemorrhage caused by puncture (n = 2), and organ failure in late-stage (n = 2). The incidence of multi-organ failure was significantly higher in the GC group than in the NGC group (33.3% vs. 11.9%, *P* = 0.003 < 0.05). The incidences of organ failure (31.4% vs. 24.9%, *P* = 0.206), organ failure lasting more than 48 h (45.8% vs. 36.2%, *P* = 0.124), peripancreatic necrotic infection (14.5% vs. 10.1%, *P* = 0.324), and proportion of SAP (32.9% vs. 28.0%, *P* = 0.371) were higher in the GC group than in the NGC group, but there was no significant difference. However, there was no significant difference in the proportion of patients using antibiotics between the two groups (51.3% vs. 46.9%, *P* = 0.463). The proportion of patients requiring advanced interventions, such as abdominal paracentesis drainage (APD), percutaneous catheter drainage (PCD), or open surgery, was not significantly different between the two groups (*P* > 0.05). The LOS in the ICU was significantly higher in the GC group than in the NGC group (3.0 ± 1.9 vs. 1.2 ± 0.7, *P* < 0.001). Pain duration, fasting time, and LOS in the hospital were higher in the GC group than in the NGC group, but there were no significant differences (*P* > 0.05). The patients in the GC group had a higher hospital cost (26,517.2 ± 2832.6 vs. 18,246.2 ± 5146.5, *P* < 0.001). These results also implied that the severity of the disease may have been higher in the GC group than in the NGC group.

**Table 2:** Main Clinical Outcomes of the Study

Characteristic	Original cohort (n = 818)		*P*	Matched cohort (n = 118)		*P*
	GC group (n = 76)	NGC group (n = 742)		GC group (n = 59)	NGC group (n = 59)	
Mortality, n (%)	3 (3.9%)	18 (2.4%)	0.676	3 (5.1%)	8 (13.6%)	0.113
Severe acute pancreatitis, n (%)	25 (32.9%)	208 (28.0%)	0.371	23 (39.0%)	33 (55.9%)	0.065
Organ failure						
Patient number, n (%)	24 (31.6%)	185 (24.9%)	0.206	20 (40.7%)	31 (52.5%)	0.041*
Duration, n (%)						
< 48 h	13 (54.2%)	118 (63.8%)	0.785	10 (50.0%)	12 (38.7%)	0.636
≥ 48 h	11 (45.8%)	67 (36.2%)	0.124	10 (50.0%)	19 (61.3%)	0.054
Organs involved, n (%)						
Single	16 (66.7%)	163 (88.1%)	0.854	13 (65.0%)	10 (32.3%)	0.486
Mutiple	8 (33.3%)	22 (11.9%)	0.003*	7 (35.0%)	21 (67.7%)	0.002*
Further interventions needed, n (%)						
APD	15 (19.7%)	139 (18.7%)	0.831	11 (18.6%)	17 (28.8%)	0.194
PCD	10 (13.2%)	72 (9.7%)	0.340	8 (13.6%)	13 (22.0%)	0.229
Minimally invasive interventions	5 (6.6%)	24 (3.2%)	0.240	4 (6.8%)	7 (11.9%)	0.342
Open operation	2 (2.6%)	11 (1.5%)	0.778	2 (3.4%)	3 (5.1%)	0.648
Antibiotic usage, n (%)	39 (51.3%)	348 (46.9%)	0.463	28 (47.5%)	29 (49.2%)	0.854
Glucocorticoids related adverse events						
Infection, n (%)	23 (30.3%)	221 (29.8%)	0.892	19 (32.2%)	18 (30.5)	0.843
Peripancreatic necrosis infection, n (%)	11 (14.5%)	75 (10.1%)	0.324	9 (15.3%)	15 (25.4%)	0.170
Gastrointestinal bleeding, n (%)	1 (1.3%)	3 (0.4%)	0.323	1 (1.7%)	1 (1.7%)	1.000
Pain duration,d	2.5 ± 1.1	1.8 ± 1.2	0.117	2.5 ± 1.1	2.9 ± 1.3	0.247
NOP duration, d	2.9 ± 1.0	2.1 ± 0.9	0.122	2.9 ± 1.1	3.3 ± 2.1	0.142
ICU LOS, d	3.0 ± 1.9	1.2 ± 0.7	< 0.001*	3.0 ± 1.8	3.4 ± 2.2	0.067
Hospital LOS, d	12.7 ± 5.4	10.3 ± 5.1	0.141	12.9 ± 5.5	16.3 ± 7.7	0.047*
Hospital costs, CNY	26,517.2 ± 2832.6	18,246.2 ± 5146.5	< 0.001*	25,348.4 ± 2512.6	32,421.7 ± 2813.3	0.002*

Abbreviation: APD, abdominal paracentesis drainage; PCD, percutaneous catheter drainage; NOP, nil per os; LOS, length of stay

*Significant difference

After matching, the patients’ clinical outcomes were reversed. The proportion of patients with organ failure (40.7% vs. 52.5%, *P* = 0.041 < 0.05) and multi-organ failure (35.0% vs. 67.7%, *P* = 0.002 < 0.05) was significantly lower in the GC group than in the NGC group. The proportion of SAP was lower in the GC group than in the NGC group, although there was not a significant difference (39.0% vs. 55.9%, *P* = 0.065). Meanwhile, mortality in the GC group was also lower than that in the NGC group, although there was not a significant difference (5.1% vs. 13.6%, *P* = 0.113). There was no significant difference in LOS in the ICU (3.0 ± 1.8 vs. 3.4 ± 2.2, *P* = 0.067) and proportion of patients using antibiotics (47.5% vs. 49.2%, *P* = 0.854) between the two groups. Patients in the GC group had significantly lower LOS in the hospital (12.9 ± 5.5 vs. 16.3 ± 7.7, *P* = 0.047 < 0.05) and cost (25,348.4 ± 2512.6vs. 32,421.7 ± 2813.3, *P* = 0.002 < 0.05) than those in the NGC group. At the same time, we found that the use of GCs did not increase the risk of gastrointestinal bleeding.

### GCs administration may facilitate fluid resuscitation in the early stage of AP

We further analyzed fluid resuscitation in the early stage of AP (within 72 h) in both groups (shown in Table 3). Before propensity score matching, patients in the GC group had significantly higher rehydration in the first 8 h than those in the NGC group (2814.8 ± 428.1 vs. 1533.4 ± 297.4, *P* < 0.001). Compared to the NGC group, patients in the GC group showed a positive balance of fluid intake and output over 72 h. After propensity score matching, there was no significant difference in rehydration volume in the first 8 h between the two groups (2774.1 ± 413.8 vs. 2711.8 ± 391.4, *P* = 0.947). Patients in the NGC group showed a positive balance of fluid intake and output over 72 h. Before matching, patients in the GC group had a significantly lower heart rate at 72 h than those in the NGC group (113.1 ± 10.9 vs. 83.7 ± 11.3, *P* < 0.001). After matching, the heart rate was no significant difference between the two groups (110.9 ± 10.7 vs. 119.4 ± 12.7, *P* = 0.374) and the patients in the GC group had a significantly lower central venous pressure than those in the NGC group (11.7 ± 2.1 vs. 16.5 ± 2.3, *P* < 0.001). However, central venous pressure was not measured in all patients. These results preferentially suggest that GCs administration may facilitate fluid resuscitation in the early stage of AP.

**Table 3:** Fluid resuscitation-related characteristics

Characteristic	Original cohort (n = 818)		*P*	Matched cohort (n = 118)		*P*
	GC group (n = 76)	NGC group (n = 742)		GC group (n = 59)	NGC group (n = 59)	
Amount of resuscitation fluids within the first 8 h, mL	2814.8 ± 428.1	1533.4 ± 297.4	< 0.001*	2774.1 ± 413.8	2711.8 ± 391.4	0.947
Total fluid balance within						
8 h, mL	2348.4 ± 401.9	1108.74 ± 259.3	< 0.001*	2270.1 ± 394.5	2318.8 ± 401.9	0.884
24 h, mL	1798.1 ± 394.8	1342.9 ± 377.4	0.022*	1763.1 ± 381.4	1790.9 ± 341.3	0.941
48 h, mL	1371.8 ± 331.4	896.7 ± 294.3	0.039*	1355.6 ± 324.7	2057.1 ± 313.1	0.012*
72 h, mL	1159.1 ± 284.2	730.1 ± 251.1	0.007*	1189.4 ± 267.4	2545.1 ± 338.7	< 0.001*
Hemodynamic and perfusion-related variables at 72 h						
Heart rate, /min	113.1 ± 10.9	83.7 ± 11.3	< 0.001*	110.9 ± 10.7	119.4 ± 12.7	0.374
CVP, cm H_2_0^#^	11.8 ± 2.1	9.3 ± 1.9	0.127	11.7 ± 2.1	16.5 ± 2.3	< 0.001*
Serum lactate, mmol/L	2.3 ± 0.9	1.2 ± 0.5	0.133	2.3 ± 0.9	2.7 ± 1.1	0.243

*Significant difference

^#^CVP, Central venous pressure. CVP was measured only in patients who had a central venous catheter. All the patients in the GC group (n = 76) had measured the CVP. In NGC group, not all patients measured CVP (n = 314, before matching; n = 57, after matching)

**Fig. 1:**
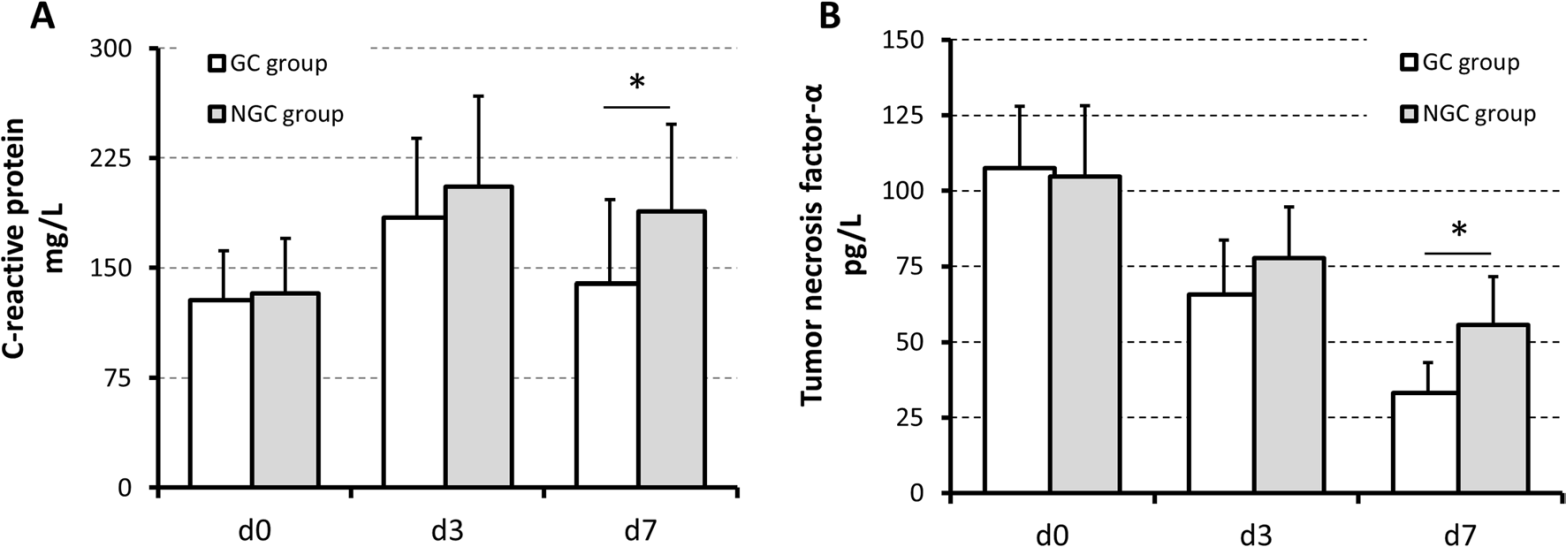
Serum levels of C-reactive protein and Tumor necrosis factor-α in the GC and NGC groups

We analyzed CRP and TNF-α levels at admission, 3 days after admission, and 7 days after admission in matched patients (shown in Fig. 1). We found that CRP increased after admission in both groups. CRP was highest 3 days after admission and decreased 7 days after admission. Compared with that in the NGC group, the CRP in the GC group was more significantly lower 7 days after admission (139.1 ± 53.2 vs. 188.5 ± 59.4, *P* = 0.013 < 0.05). In addition, TNF-α decreased after admission in both groups. TNF-α in the GC group also decreased more significantly 7 days after admission than in the NGC group (33.1 ± 10.1 vs. 55.7 ± 15.7, *P* = 0.027 < 0.05).

## Discussion

This retrospective research confirms that patients with AP can benefit from the utilization of GCs at the early phase of AP. The mortality, prevalence of multi-organ failure, hospital LOS, and costs were significantly reduced without increasing the incidence of gastrointestinal bleeding.

The role of GCs in the treatment of AP has been studied for a long time [15]. They have been considered a routine treatment for autoimmune pancreatitis [24]. However, the effects of GCs in the treatment of AP are controversial. On the one hand, to the best of our knowledge, Stephenson et al. first reported the benefit of GCs in human haemorrhagic AP in 1952 [14]. Several supporting case reports and clinical studies have been reported since then. For example, a recent study indicated that dexamethasone combined with Chinese herbal decoction decreases the risk of developing ARDS in patients with SAP [25], and a meta-analysis including six Chinese trials showed that corticosteroids might improve outcomes in patients with SAP [26]. GCs also show promising therapeutic effects in many animal models of AP, including ethionine-induced pancreatitis in rabbits, diet-induced necrotizing pancreatitis, and necrohaemorrhagic pancreatitis induced by the retrograde injection of Na-taurocholate into the pancreatic duct in mice, canines, and porcine[16, 17, 27–30]. On the other hand, there are several randomized controlled trials, and a meta-analysis showed no statistically significant benefit of prophylactic GCs for the prevention of post-endoscopic retrograde cholangiopancreatography (ERCP) pancreatitis, one sort of AP [31]. Meanwhile, GCs are also thought to potentially trigger AP [32].

In our opinion, the effect of GCs in the treatment of AP needs to be re-evaluated. More quality studies are required to investigate whether GCs are beneficial in the treatment of AP.

First, the core pathophysiological process in the early stages of AP is uncontrolled inflammation [4]. Activation of inflammatory cascades induces a systemic inflammatory response and impairs organ functions [33]. GCs are almost the most widely prescribed anti-inflammatory drugs [34]. GCs play an anti-inflammatory role through induction of the synthesis of anti-inflammatory proteins and through repression of proinflammatory transcription factors, such as NF-κB [35], which are also activated during the early stages of AP [36].

GCs are beneficial in a variety of inflammatory diseases. For example, a recent meta-analysis showed that methylprednisolone treatment could accelerate the resolution of ARDS, which was also common in SAP, improving a broad spectrum of interrelated clinical outcomes [11]. In vivo, dexamethasone also showed a protective effect on acute kidney injury by preventing microvascular endothelial glycocalyx degradation initiated by TNF-α during SAP [27]. Furthermore, GCs have been shown to exhibit protective effects on systemic inflammatory responses induced by burns, infections, or even COVID-19 [10, 13]. Thus, theoretically, GCs have a potential therapeutic effect on AP.

Second, a study showed that cyclooxygenase-2 (COX-2) inhibitors can prevent AP from becoming severe recently [37]. This is the first report to demonstrate the effect of COX-2 inhibitors on the treatment of AP in humans. COX-2 inhibitors exert inflammatory inhibitory effects by blocking the production of prostaglandins from arachidonic acids [38]. COX-2 is a highly inducible enzyme and accumulates promptly in the presence of inflammation [39]. The anti-inflammatory properties of GCs are attributed in part to their interference with prostaglandin synthesis through cyclooxygenase [40]. This evidence also implies a possible role for GCs in the treatment of AP.

Third, the evidence that GCs have no prophylactic effect in post-ERCP pancreatitis does not disclaim that GCs have no therapeutic effect in AP. The occurrence of post-ERCP pancreatitis is closely associated with repeated intubation, intubation into the pancreatic duct, and longer ERCP procedures [41]. All of these factors are physically related to pancreatic duct insult or hypertension during the process of ERCP. Placement of pancreatic ductal stents after ERCP has also been shown to be effective in reducing the incidence of post-ERCP pancreatitis [42]. ERCP is also the standard treatment for pancreatitis due to biliary tract disease. Prophylactic GCs may not alleviate pancreatic duct insult or hypertension. Moreover, post-ERCP pancreatitis is usually mild or moderately severe and presents as a self-limiting process. The therapeutic effect of GCs in post-ERCP pancreatitis remains little studied until now.

Fourth, intravenous fluid resuscitation is considered the cornerstone of management in AP [43]. It is generally recommended in all patients with AP, despite the optimal rate, type, and goal of fluid resuscitation being controversial [44]. In a variety of inflammatory diseases, inflammatory cascades are thought to induce endothelial activation and capillary leakage, leading to circulatory collapse and shock [45]. GCs could preserve endothelial integrity through upregulation of junctional proteins such as occludin, claudin-5, and VE-cadherin and downregulation of matrix metalloproteinase-9 and alleviate hemodynamic disturbances [46, 47]. For example, low-dose GCs are considered to be associated with a faster reversal of shock and a short duration of mechanical ventilation in septic shock [48]. In patients undergoing cardiac surgery, GCs also reduce proinflammatory cytokine release, slow leukocyte migration, and decrease capillary leakage associated with cardiopulmonary bypass [49]. Moreover, in vivo, GCs have been shown to protect the renal microvascular endothelium and intestinal capillary endothelium during SAP [27]. These studies hint that GCs may fulfil a similar effect in the fluid resuscitation of AP.

However, GC-induced pancreatitis has only been reported in individual cases [32, 50]. There is a lack of large-scale, high-quality evidence studies, and the mechanism of GC-induced pancreatitis remains unclear. GC-induced pancreatitis is currently thought to be correlated with high doses and long-term use and may be idiopathic [51].

The adverse effects of GCs are still noteworthy. Secondary infections, osteoporosis, wound healing difficulty, and gastrointestinal bleeding are the most concerning adverse effects of GCs therapy. GCs-induced immunosuppression is the predominant cause of secondary infections [52]. The utilization of a high dose of GCs could induce osteoporosis and eventually induce femoral head necrosis [53]. There is evidence supporting that using higher-dose GCs was an independent risk factor for bleeding [54]. Thus, patients with AP at low risk of infection were included in this study. Studies have confirmed that the risk of infection is low in the early stages of hypertriglyceridemic AP and that prophylactic antibiotic use is avoidable [55].

In this study, our results showed that the administration of GCs during the treatment of AP facilitated fluid resuscitation and reduced mortality, the prevalence of multi-organ failure, and hospital costs. At the same time, the administration of GCs during the early stage of AP did not increase the risk of infection or gastrointestinal bleeding. These data provide preliminary evidence that early GCs use is effective and relatively safe in the treatment of AP.

However, this study is retrospective, and there were some missing case details. Even though we used PSM analysis, inconsistencies in the baseline and bias could still be possible. In addition, in this study, the criterion was whether GCs should be used in the treatment of AP. We did not distinguish well between the types or amounts of GCs use or the duration of GCs use, which might also influence the therapeutic outcomes.

In summary, this retrospective study presents preliminary confirmation of the beneficial effect of GCs in the treatment of AP. More high-quality prospective studies are needed in the future.

## Data Availability

All datasets used and analyzed during the current study are available from the corresponding author upon reasonable request.
